# Green Synthesis of Iron Nanoparticles Using an Aqueous Extract of Strawberry (*Fragaria* × *ananassa* Duchesne) Leaf Waste

**DOI:** 10.3390/ma17112515

**Published:** 2024-05-23

**Authors:** Małgorzata Góral-Kowalczyk, Elżbieta Grządka, Jolanta Orzeł, Dariusz Góral, Tomasz Skrzypek, Zbigniew Kobus, Agnieszka Nawrocka

**Affiliations:** 1 Department of Agricultural Forestry and Transport Machines, Faculty of Production Engineering, University of Life Sciences in Lublin, 28 Głęboka Street, 20-612 Lublin, Poland; malgorzata.goral-kowalczyk@up.lublin.pl; 2 Institute of Chemical Sciences, Faculty of Chemistry, Maria Curie-Skłodowska University, M. Skłodowskiej-Curie 3 Sq., 20-031 Lublin, Poland; elzbieta.grzadka@mail.umcs.pl (E.G.); jolanta.orzel@mail.umcs.pl (J.O.); 3 Department of Biological Bases of Food and Feed Technologies, Faculty of Production Engineering, University of Life Sciences in Lublin, 28 Głęboka Street, 20-612 Lublin, Poland; 4 Department of Biomedicine and Environmental Research, Institute of Biological Sciences, Faculty of Medicine, The John Paul II Catholic University of Lublin, Al. Racławickie 14, 20-950 Lublin, Poland; tomasz.skrzypek@kul.pl; 5 Department of Technology Fundamentals, Faculty of Production Engineering, University of Life Sciences in Lublin, 28 Głęboka Street, 20-612 Lublin, Poland; zbigniew.kobus@up.lublin.pl; 6 Department of Physical Properties of Plant Materials, Institute of Agrophysics, Polish Academy of Sciences, Doświadczalna 4, 20-290 Lublin, Poland; a.nawrocka@ipan.lublin.pl

**Keywords:** green chemistry, nanoparticle synthesis, iron nanoparticles, strawberry leaf, polyphenols, flavonoids

## Abstract

In this study, we analysed the potential use of dried strawberry leaves and calyces for the production of nanoparticles using inorganic iron compounds. We used the following iron precursors FeCl_3_ × 6H_2_O, FeCl_2_ × 4H_2_O, Fe(NO_3_)_3_ × 9H_2_O, Fe_2_(SO_4_)_3_ × H_2_O, FeSO_4_ × 7H_2_O, FeCl_3_ anhydrous. It was discovered that the content of polyphenols and flavonoids in dried strawberries and their antioxidant activity in DPPH and FRAP were 346.81 µM TE/1 g and 331.71 µM TE/1 g, respectively, and were similar to these of green tea extracts. Microimages made using TEM techniques allowed for the isolation of a few nanoparticles with dimensions ranging from tens of nanometres to several micrometres. The value of the electrokinetic potential in all samples was negative and ranged from −21,300 mV to −11,183 mV. XRF analyses confirmed the presence of iron ranging from 0.13% to 0.92% in the samples with a concentration of 0.01 mol/dm^3^. FT-IR spectra analyses showed bands characteristic of nanoparticles. In calorimetric measurements, no increase in temperature was observed in any of the tests during exposure to the electromagnetic field. In summary, using the extract from dried strawberry leaves and calyxes as a reagent, we can obtain iron nanoparticles with sizes dependent on the concentration of the precursor.

## 1. Introduction

Nanoparticles of iron and its oxides are commonly used in many areas, ranging from industry and medicine to agriculture and environmental protection. This is due to their unique properties and low production costs. From a practical point of view, the most important applications of nanoparticles include catalysis, photomagnetism, the magneto-optic effect, data storage, sensor manufacturing, inkjet printers, high-frequency and radio frequency devices, dietary supplements production, chemical analysis, as well as tissue imaging and drug carriers [1,2]. Nanoparticles of iron and iron oxides are non-toxic, thanks to which they can be used in the food industry [3]. In addition, iron nanoparticles are characterised by good dimensional stability and high thermal and electrical conductivity, attributes increasingly important for the development of medicine [4].

In recent years, a number of articles on green chemistry have appeared. Such great interest in this field results from the possibility of designing alternative, safer, energy-efficient, and less toxic methods for the nanoparticle synthesis. These methods have been associated with a rational use of various substances in nanoparticle products and synthetic methods [5,6,7,8]. A particular advantage is that the production of nanoparticles in this way does not require specialised technical knowledge or well-equipped and dedicated laboratories. Besides, nanoparticles can be easily obtained by conducting experiments at room temperature [9].

Green chemistry uses various biological materials, such as fungi, bacteria, viruses, bacteriophages, algae and plants [3]. Chemical reduction is widely used in nanoparticle synthesis. The general methodology and materials used in nanoparticle synthesis include reducing agents, safeners, solvents and metal salt precursors. Most methods use highly reactive reducing agents contained in biological material, such as amino acids, aldehydes or flavonoids [10]. These ingredients are non-toxic, biodegradable and may function as reducing and encapsulating agents, thus promoting the formation of nanoparticles while inhibiting their agglomeration.

Plants, or their parts, are easy to obtain and inexpensive to grow, making them the preferred materials used in green chemistry. There have been several studies focusing on testing and evaluating plants to obtain metal nanoparticles [6,10,11]. The use of plant extracts in obtaining nanoparticles involves mixing them with a solution of metal salts at room temperature. Typically, the reaction lasts a few minutes and as a result, metals are transformed from their mono- or divalent oxidation states to zero oxidation states, changing the colour of the system. Due to different concentrations of active biochemical compounds, the use of different parts of the plant may affect the morphology of the synthesised nanoparticles. The mechanism of metal ion reduction to gold and silver nanoparticles by plant extracts has been described in several studies, including Guo et al. [12] and Zuhrotun et al. [13]. The authors explain that metal ions are complexed by phenolic OH groups and then reduced to metal. Another property that enables the formation of nanoparticles is the reduction potential. This potential is different for each metal and significantly affects the reaction rate. If a positive reduction potential is larger, the metal precursor can be reduced faster [10].

Strawberry (*Fragaria* × *ananassa* Duchesne) is one of the most widely cultivated fruit crops worldwide. It is eagerly consumed due to its taste and nutritional value. The fruits are rich in polyphenols, phenolic acids and flavanols. A high intake of products containing significant amounts of the above compounds is strongly correlated with a lower incidence of some chronic diseases related to the cardiovascular system, obesity and Alzheimer’s disease [14,15]. In addition to the fruits, the less popular strawberry leaves also deserve to be noted. They have antioxidant properties and contain over 20 different phenolic compounds, including health-promoting ellagitannins [16,17,18]. It has been shown that the leaves have antioxidant capacity higher than the fruits [19]. Strawberry leaves, like those of other fruit plants, are mostly considered waste material after harvesting [18]. According to FAO data, worldwide strawberry production in 2020 amounted to 8.9 million tonnes [20], resulting in significant volumes of waste.

The utilisation of strawberry leaf extract is a promising avenue to produce new, healthy food products, food additives, nanoparticles and the effective use of biomass waste, such as leaves. Leaves are, by far, the plant part most frequently used for the synthesis of iron nanoparticles. The leaf extracts from tea (*Camellia sinensis*), eucalyptus (*Eucalyptus globulus*), oak (*Quercus* spp.) and pomegranate (*Punica granatum*) are used most often [3,21,22,23]. This study aimed to analyse the possibility of using dried strawberry leaves and calyces to produce nanoparticles with the use of inorganic iron compounds.

## 2. Materials and Methods

### 2.1. Materials

The research material consisted of cut leaves (*Fragariie folium*) and calyces of strawberries of various varieties grown in Poland. The dried raw material was stored in a tight package without access to sunlight. The iron precursors used were FeCl_3_ x 6H_2_O (Chempur, Piekary Śląskie, Poland), FeCl_2_ × 4H_2_O (POCH, Gliwice, Poland), Fe(NO_3_)_3_ × 9H_2_O (Chempur, Piekary Śląskie, Poland), Fe_2_(SO_4_)_3_ × H_2_O (POCH, Gliwice, Poland), FeSO_4_ × 7H_2_O (Chempur, Piekary Śląskie, Poland) and FeCl_3_ anhydrous (Chempur, Piekary Śląskie, Poland).

### 2.2. Total Content of Polyphenols and Flavonoids

To prepare the extract, 2 g of ground sample of strawberry leaves and calyces was mixed with 30 mL of methanol in a 100 mL conical flask. Then, the mixture was stirred using a magnetic stirrer and heated to a temperature of 30 °C. After two hours of stirring, the extract was collected in a separate flask and the raffinate was mixed with another 30 mL of methanol. After two hours of extraction, using a magnetic stirrer, the extract was collected and mixed with the previous portion of the extract and centrifuged at 6500 rpm for 15 min.

The total phenolic content was determined according to the methodology described by Kobus et al. [24] with a slight modification. A 0.06 mL amount of extract was mixed with 2 mL of Folin–Ciocalteu reagent in a 25 mL flask. After three minutes, 2 mL of sodium carbonate was added and the volume was topped up to 25 mL with distilled water. After the next 30 min of incubation in darkness, the absorbance at a wavelength of λ = 760 nm was measured. Results were expressed as mg gallic acid equivalent per 1 g of dry matter of strawberry leaves and calyces.

The total flavonoid content was determined according to the methodology described by Aryal et al. [25] with a slight modification by Kobus et al. [24]. Firstly, 0.3 mL of the sample extract was combined with a 2% AlCl_3_ × 6H_2_O solution (in methanol) in a volume ratio of 1:1. The obtained mixture was then topped up to a volume of 10 mL with distilled water and then subjected to 10 min incubation under dark conditions and at room temperature. After incubation, the absorbance of the mixture was assessed at a wavelength of λ = 430 nm. Absorbance was measured with a UV 1800 spectrophotometer (Shimadzu, Kyoto, Japan). Flavonoid concentrations were assessed in relation to a calibration curve prepared with quercetin and reported as mg quercetin equivalent per 1 g dry matter (mg QE g^−1^ dry matter). The measurement was performed in three runs for each sample.

### 2.3. Determination of Antioxidant Activity (DPPH and FRAP Assays)

The antioxidant activity of the tested solutions was determined using DPPH and FRAP reagents (Merck KGaA, Darmstadt, Germany). A total of 60 μL of the extract and 5.8 mL of 6 × 10^−5^ M DPPH were mixed in a 10 mL flask and after 30 min of incubation in darkness, the absorbance was measured at a wavelength of λ = 516 nm. A total of 60 μL of the extract and 5.8 mL of FRAP solution were mixed in a 10 mL flask and after 10 min of incubation in the dark at 37 °C, the absorbance was measured at a wavelength of λ = 593 nm. In both cases, the obtained results were expressed as Trolox equivalent in μg^−1^ g dry matter. The measurement was performed in three runs for each sample.

### 2.4. Nanoparticle Synthesis

Iron nanoparticles were obtained through green synthesis. For this purpose, dried leaves and calyxes with a total weight of 50 g were crushed with a mortar and pestle. The obtained raw material was combined with 500 mL of distilled water. The suspension was heated in a water bath (IKA, Warsaw, Poland) at 65 °C for 1 h. After cooling to ambient temperature, the obtained extract was filtered. The previously prepared solutions of iron precursors were added to the prepared extract and 9 samples were prepared according to Table 1.

Additionally, a control sample was prepared containing pure extract from strawberry leaves and calyces, without the precursors. The obtained solutions were transferred to sterile 2 mL Eppendorf tubes, stirred for 30 min and then centrifuged twice (6000 rpm, time 10 min, temperature 6 °C) (MPW–352 R, MPW Med. Instruments, Warsaw, Poland) each time washing the samples with distilled water. Samples intended for further testing were stored at 6 °C in a Q-Cell 140 refrigerator (Pol-lab, Wilkowice, Poland).

### 2.5. Zeta Potential Analysis and Particle Size Distribution

The zeta potential was analysed with a NanoZS Zetasizer (Malvern Instruments, Ltd., GB, Malvern, UK) which uses laser Doppler velocimetry to determine electrophoretic mobility. The electrophoretic mobility of the sample was recalculated into the zeta potential using the Smoluchowski equation. The universal dip cell including the electrode assembly with palladium electrodes with 2 mm spacing was used for the measurements. The experiment was carried out in PCS1115 cuvettes. The same apparatus was used for particle size distribution measurements using the DLS technique. DLS estimates the hydrodynamic diameter (mean diameter, z-average) and the polydispersity index (PI) as a measure of particle size distribution. The mean diameter and PI of the analysed samples were determined by calculating the average of six measurements, at an angle of 173°, in 10 mm diameter disposable plastic polystyrene cuvettes. Histograms with the particle size distributions were also recorded. All the experiments were performed at 25 °C. Each measurement was repeated six times and the values were averaged.

### 2.6. X-ray Fluorescence

Energy-dispersive X-ray fluorescence spectrometry (XRF) was used to analyse the content of heavy metals in the samples. This device enables the analysis of solid samples that occur in trace amounts, i.e., below 1 mg/kg, in lower density matrices. The XRF is equipped with a 600 W X-ray tube with a gadolinium anode, powered by a voltage of up to 100 kV. To measure fluorescence radiation, a germanium detector (with a beryllium window) with ultra-high resolution (below 140 eV) cooled with liquid nitrogen was used. The research material in the form of solutions was analysed in its entirety (direct measurement). After weighing, the samples were placed in measuring cups with supporting foil (Myler foil). All measurements were performed with the standard-free Auto Quantify method using the ED-XRF Epsilon 5 (Panalytical, Almelo, The Netherlands) and the Genie 2000 software.

### 2.7. Transmission Electron Microscope (TEM) Micrographs

The samples were applied to formvar grids and examined using a TEM microscope (Zeiss LIBRA, Oberkochen, Germany) at an accelerating voltage of 80 kV, with 50 kx magnification.

### 2.8. Collection of Fourier Transform Infrared (FT-IR) Spectra

The FT-IR spectra were recorded with a Nicolet 6700 FT-IR spectrometer (Thermo Scientific, Madison, WI, USA) equipped with a diamond ATR attachment. The samples were analysed in powder form. Before FT-IR analyses, aqueous samples of Fe nanoparticles were freeze-dried for 24 h. The FT-IR spectra were recorded between 4000 and 400 cm^−1^ at 4 cm^−1^ intervals. Each spectrum resulted from 128 scans to obtain optimal signal-to-noise ratios. Each spectrum was corrected with a linear baseline using OMNIC software (v.8.2, Thermo Fischer Scientific Inc., Madison, WI, USA). The analysed spectra were averaged over five registered spectra.

### 2.9. X-ray Diffraction Analysis (XRD)

The measurements carried out were performed according to the following method in triplicate. The powder diffraction data were collected at room temperature using an Empyrean diffractometer with a PIXcel3D detector (PANalytical, Almelo, The Netherlands) and monochromated Cu–Kα radiation (λ = 1.54184 Å), in the 2θ range of 4.7–50°.

### 2.10. Magnetic Properties

The measurements included a control sample containing pure plant extract and three samples with concentration of 0.01 mol/dm^3^ with different iron precursors: 111 (with FeCl_3_ × 6H_2_O), 333 (with FeCl_2_ × 4H_2_O) and 666 (FeSO_4_ × 7H_2_O). Before the analyses, the nanoparticles were vortexed and then exposed to ultrasound for a brief moment. AMF was generated with the magneTherm™ system (nanotherics Ltd., Staffordshire, UK). A standard system was used, containing solenoid coils with 17 turns and 9 turns, capable of generating AMF up to 25 mT within the range of 100 kHz–1 MHz. Each sample was exposed to an electromagnetic field for 600 s and then cooled for another 300 s. Pico M™ with OTG-MPK 5 optical sensor system (Opsens, Quebec, QC, Canada) was used for real-time temperature monitoring.

### 2.11. Statistical Analysis

Significant differences between particular groups were found using the Student *t*-test and variance analysis (ANOVA). The Pearson correlation coefficient was also performed. All statistical analyses were performed using the open source software R, version 4.3.2 [26].

## 3. Results and Discussion

### 3.1. Total Polyphenolic/Flavonoid Content and Antioxidant Activity

Catalytic reactions are the pillar of green chemistry. Thanks to their selectivity, catalysts contribute to the reduction of waste in manufacturing processes, thus reducing activation energy consumption. The processes that involve them can be run in easier-to-achieve conditions. Various factors (e.g., pH, pressure, temperature, type of solvent) influence “green” synthesis techniques. However, a key role is played by phytochemicals that can be found in plant extracts (roots, leaves, stems, fruits). These are the following: ascorbic acids, phenols, carboxylic acids, terpenoids, amides, flavones, aldehydes, ketones, etc. [27,28]. These components reduce metal salts into metal nanoparticles [29]. Phytochemicals contained in plants include flavonoids and polyphenols. They have strong antioxidant properties. They play a major role in the reduction of metal ions into nanoparticles [30]. There are many studies revealing that strawberries contain phytochemicals with anti-inflammatory or anticancer properties [31,32]. It is known that the leaves of berry-producing plants have a high content of phenolic compounds, much higher than fruits [19]. There are also known methods of obtaining metal nanoparticles using the extract from strawberry fruits [33] and leaves [34]. The total content of polyphenols was tested with a view of using waste raw material, such as strawberry leaves and calyces, in the reduction of iron salts. The obtained results are presented in Table 2.

According to Kårlund et al. [16], the total polyphenol content of polka strawberry leaves, grown in Finland, was 81.15 ± 0.64 (mg GAE/1 g). Taking into account that in our research, we used leaves and calyces of several varieties of strawberries grown in Poland, the polyphenol results obtained do not differ significantly from those obtained by Kårlund et al. [16]. Similarly, according to Cvetković et al. [35], the flavonoid content in the leaves of the Senga sengana and Mount Everest varieties ranged from 6.2 mg QE/1 g to 7.74 mg QE/1 g depending on the extraction method and variety. The measured flavonoid content in leaves and calyces in our research was 4.66 mg QE/1 g. In the case of DPPH and FRAP, the obtained values of 346.81 µM TE/1 g and 331.71 µM TE/1 g, respectively, indicate high antioxidant activity. A popular raw material for obtaining iron nanoparticles using green chemistry is green tea leaf extract. It has been used in many studies [36,37,38,39]. According to Cho et al. [40], the values of green tea leaf extract in DPPH and FRAP assays were 132.3 ± 13.5 µM TE/1 g and 160.0 ± 16.7 µM TE/1 g, respectively. Compared to the values obtained in the case of strawberry leaves, they were lower, which proves that the raw material used in this research is very promising. It should be emphasised that the basic mechanism of iron NP formation, including nucleation and particle growth, by plant extracts is not fully understood. However, research shows that plants with high concentrations of phenolic compounds are the best choice for the production of iron nanoparticles using green chemistry [41].

### 3.2. Iron Nanoparticle Synthesis

**Figure 1:**
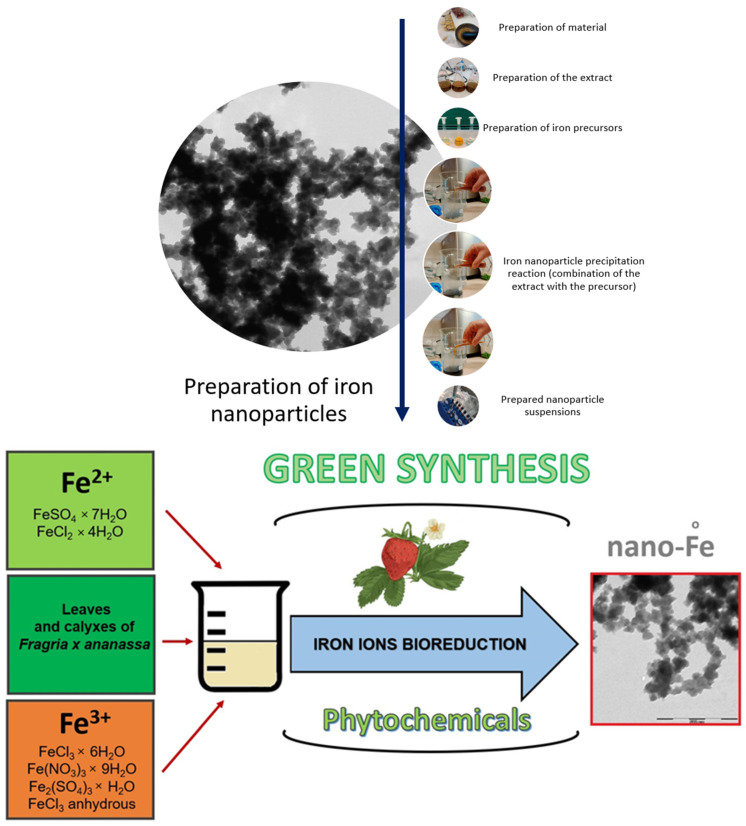
General scheme for the synthesis and bioreduction of iron nanoparticles.

As already mentioned, an extract from strawberry leaves and calyxes was used to produce iron nanoparticles. The reduction of iron ions to iron particles occurred after mixing iron salts (Table 1) with the plant extract. The colour of the reaction mixture containing the extract and iron salts changed from the colour typical of the dissolved salt to green-black and black (depending on the precursor used) at 20 °C, as shown in Figure 1.

The colour change resulted from the reduction of iron ions, which may indicate the formation of iron nanoparticles. The formation and bioreduction mechanism of iron precursor compounds in the presence of phytochemicals from the extract of dried leaves and calyxes of strawberries are shown in Figure 1.

A similar reaction has been observed in many studies shown in Table 3 and additionally in publications by Wang [42], Bolade et al. [43] and Jain et al. [44].

### 3.3. Microscopic Imaging

Due to difficulties in sample preparation and micrograph SEM interpretation, the same and subsequent samples were analysed using a transmission electron microscope. The images obtained from the TEM microscope were clearer and allowed for the isolation of individual particles, and the assessment of their shape. Moreover, the images show darker regions indicating metallic iron and lighter regions representing iron oxide phases [49].

**Figure 2:**
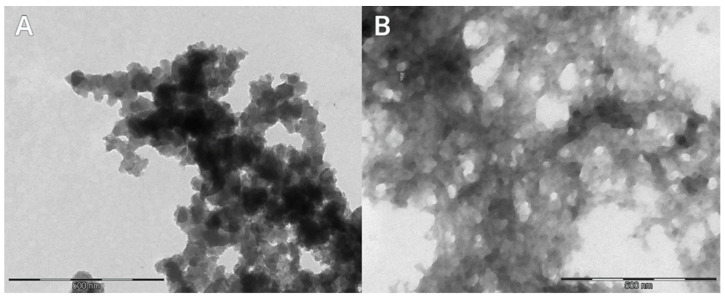
Transmission electron microscope (TEM) images of sample 11 with a concentration of 0.1 mol/dm^3^ and sample 111 (**A**) with a concentration of 0.01 mol/dm^3^ with FeCl_3_ × 6H_2_O (**B**).

**Figure 3:**
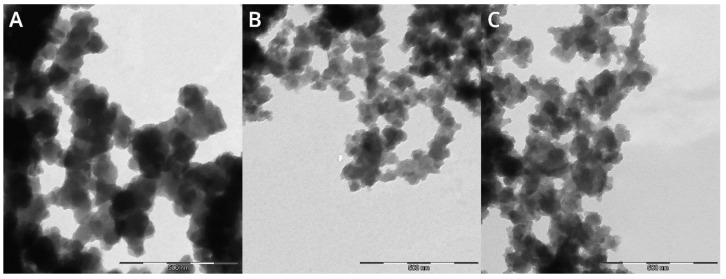
Transmission electron microscope (TEM) images of samples with concentration 0.01 mol/dm^3^: 333 with FeCl_2_ × H_2_O (**A**), 555 with FeCl_3_ anhydrous (**B**) and 666 with FeSO_4_ × 7H_2_O (**C**).

The particles obtained in samples 1 and 2 were mostly spherical, and some of them had a triangular or polygonal shape. The particle dimensions were in the range of 129–182 nm. In sample 11, individual nanoparticles with an average size of 60 nm with an irregular shape and clearly darker fields, as well as particles measuring above 100 nm, were observed. Sample 111 was characterised by the smallest spherical particles with a visibly lighter colour. The particle diameter ranged from 13 nm to 34 nm. Many of them formed aggregates that were difficult to identify (Figure 2). Sample 333 was characterised by the largest particles, when compared to other samples, with a concentration of 0.01 mol/dm^3^. The nanoparticles identified in the images ranged in size from 70 nm to 145 nm (Figure 3A).

Isolation of individual nanoparticles was very difficult due to their strong aggregation. The particles in sample 555 had an oblong shape with clearly defined darker regions. Their average size ranged from 29 nm to 52 nm (Figure 3B). Particles from sample 666 were characterised by a very variable shape, from nanocubes and spheres to triangles with irregular edges. Their average size ranged from 50 to 78 nm (Figure 3C). Darker regions were clearly noticeable. It should be noted that the shape of the iron nanoparticles was similar to the shape of iron nanoparticles obtained in the studies of Nadagouda et al. [50], Sharma et al. [51] and Jain et al. [44].

### 3.4. Particle Size Distribution and Zeta Potential

**Figure 4:**
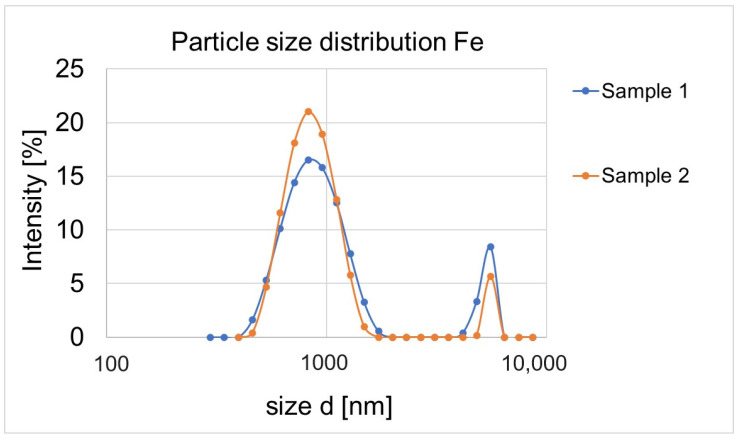
Particle sample distribution in sample 1 with a concentration of 0.5 mol/dm^3^ and FeCl_3_ × 6H_2_O and sample 2 with a concentration of 0.5 mol/dm^3^ and Fe_2_(SO_4_)_3_ × H_2_O.

In the first stage of the research, six samples (1, 2, 11, 22, 33, 44) were prepared at concentrations of 0.5–0.1 mol/dm^3^, which were analysed in terms of particle size and zeta potential. Based on the obtained results, it was claimed that the average particle size in sample 1 is 1342.78 ± 76.87 nm, and in sample 2 it is 1257.33 ± 88.18 nm. The smallest particles observed in sample 1 measured 291 nm, and in sample 2 396 nm (Figure 4).

**Figure 5:**
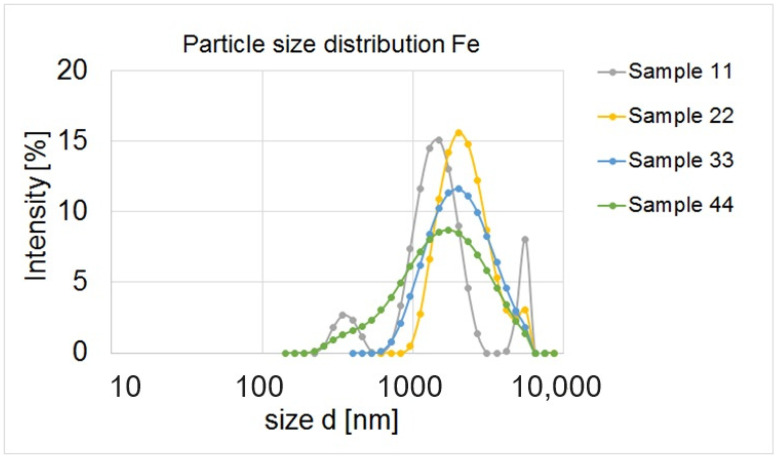
Particle sample distribution in samples in concentration 0.1 mol/dm^3^: 11 (with FeCl_3_ × 6H_2_O), 22 (with Fe_2_(SO_4_)_3_ × H_2_O), 33 (with FeCl_2_ × 4H_2_O) and 44 (with Fe(NO_3_)_3_ × 9H_2_O).

A similar particle size scale was obtained in samples 11, 22, 33 and 44. The smallest particles were observed in samples 44 (142 nm) and 11 (164 nm) (Figure 5).

**Figure 6:**
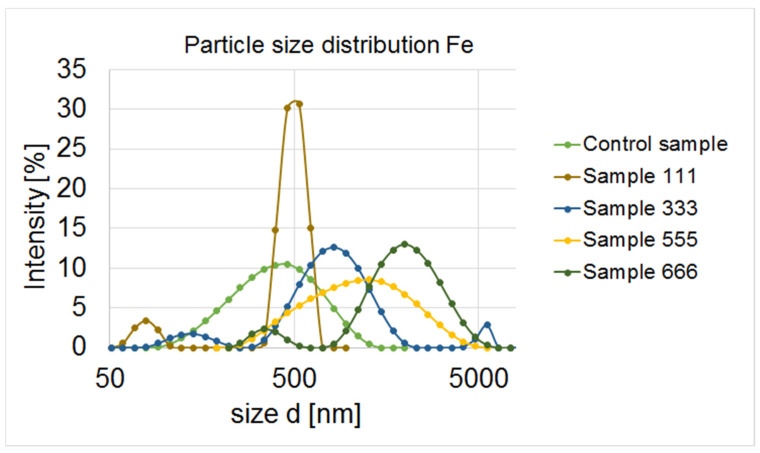
Particle sample distribution in control sample and samples in concentration 0.01 mol/dm^3^: 111 (with FeCl_3_ × 6H_2_O), 333 (with FeCl_2_ × 4H_2_O), 555 (with FeCl_3_ anhydrous) and 666 (with FeSO_4_ × 7H_2_O).

Analysing the graph of the size distribution of nanoparticles, we can assume that the nanoparticles in sample 11 form aggregates that can be observed on the graph in the form of three peaks. Similarly, two peaks were obtained by Goldstein and Greenlee [52]. They found that the second peak indicates agglomeration of nanoparticles. Sample no. 44 is very unstable in terms of particle size, and most likely they are not nanoparticle aggregates, but micro-scale particles. In sample 22, only very large particles ranging in size from 531 nm to 8630 nm can be seen. Nanoparticles are defined as materials with at least one dimension in the size range of 1–100 nm [53]. The obtained values were much higher than expected and did not fall within the range reserved for the size of nanoparticles. Therefore, further samples were prepared (in lower concentrations and with other precursors). Then, they were analysed for particle size distribution. The obtained results are shown in Figure 6.

A decision was made to repeat sample 11 with a lower concentration of the precursor (0.01 mol/dm^3^), thus creating sample no. 111. Dilution of the sample led to the formation of nanoparticles with a size of 37.8 nm to 91.3 nm. In this sample, an aggregate of nanoparticles ranging from 342 to 615 nm was also likely observed. No particles larger than 955 nm were observed. Then, a decision was made to additionally dilute sample 33 further, creating sample 333 with a concentration of 0.01 mol/dm^3^ of the precursor. Based on the particle size distribution of this sample, we found nanoparticles with a size of 50.7–90.3 nm and a size of 342–615 µm, which may constitute nanoparticle aggregates. Due to the correct size of the nanoparticles, we wanted to test solutions with previously unused precursors, i.e., anhydrous iron (III) chloride and hydrated iron (II) sulphate at a concentration of 0.01 mol/dm^3^. Unfortunately, in terms of particle size distribution, the trials performed were worse compared to samples 111 and 333. In both samples 555 and 666, no particles smaller than 100 nm were observed. Sample 555 was very heterogeneous in terms of the particle size. In sample 666, an aggregate with a particle size of 255–531 nm was likely observed. The prepared solutions were compared with a control sample containing pure strawberry leaf extract.

Factors that affect zeta potential are solution pH, ionic strength and the types of ionic species present in the system [54]. The electrokinetic potential values were calculated for all prepared samples. Their values were negative in all samples and ranged from −21.300 mV to −11.183 mV. This proves that the particles dispersed in suspensions are negatively charged. The highest potential values were observed in the pure extract and sample 22 with the addition of Fe_2_(SO_4_)_3_ at a concentration of 0.1 mol/dm^3^. The lowest potential values were recorded in samples 33, 44, 111 and 333. An absolute zeta potential value >30 mV generally indicates that the particles are electrostatically stable [52]. In the case of the analysed systems, it can be concluded that samples 33, 333 and 111 are the most stable, while sample 22 is the least stable. Low stability means a greater tendency to aggregation, which may explain the discrepancies between the particle size distribution analysis and microscopic data.

The Pearson correlation coefficient for the dependence of the concentration on the zeta potential value was 0.13, which indicates a negligible correlation. We also performed a single-criterion analysis of variance (ANOVA), taking into account whether the added precursor had a significant effect on the zeta potential value. The obtained results indicate that no significant differences were found in the case of samples with the addition of FeCl_3_ × 6H_2_O, FeCl_2_ × 4H_2_O, Fe(NO_3_)_3_ × 9H_2_O, FeCl_3_ anhydrous and FeSO_4_ × 7H_2_O. Only the samples with the addition of Fe_2_(SO_4_)_3_ × H_2_O and the control sample showed a significantly higher zeta potential value (Table 4). A negative value of the zeta potential was also demonstrated by Iqbal et al. [55] synthesising IONPs using *Rhamnella gilgitica* leaf extract and Kanagasubbulakshmi and Kadirvelu [56] examining the properties of iron oxide nanoparticles using *Lagenaria siceraria*.

### 3.5. Measurement of Elemental Composition

Based on the microscopic results and particle size distribution, we decided to carry out XRF analyses only for samples with concentrations between 0.1 mol/dm^3^ and 0.01 mol/dm^3^. Detailed results are presented in Table 5.

The presence of iron was found in all samples. The measurement results indicate that the iron concentration in the samples was variable and ranged from 0.09% to 1.31% in samples with a concentration of 0.1 mol/dm^3^ and from 0.13 to 0.9 in samples with a concentration of 0.01 mol/dm_3_. The iron content is higher in all samples compared to the control containing only pure leaf extract. Potassium, iron, calcium, phosphorus, silicon and sulphur naturally occur in strawberry leaves. Chlorine, on the other hand, may come from alkaloids [57]. The results of the XRF analysis allow for determining the general total iron content, and not the resulting compound [58]. The amount of an element present in the tested object depends on the intensity of the detected signal at its characteristic energy value.

### 3.6. FTIR and XRD Analysis

**Figure 7:**
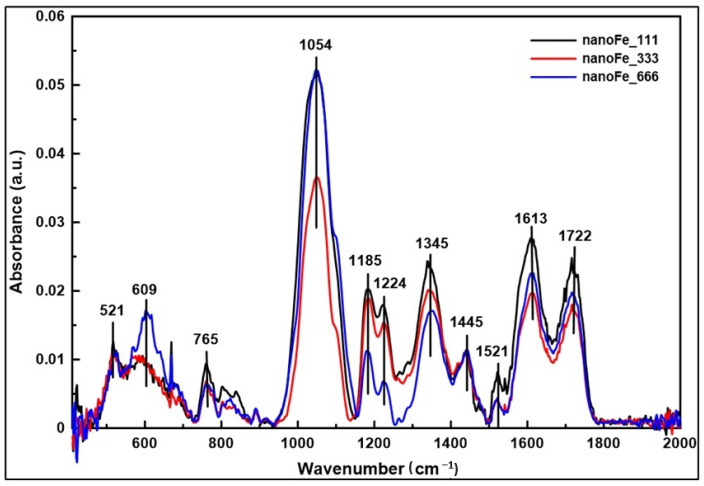
FT-IR spectra of Fe nanoparticles.

The FT-IR bands at 521 and 609 cm^−1^ can be assigned to the Fe nanoparticles (Figure 7). Bands characteristic for Fe nanoparticles in a similar spectra region were observed by Hwang et al. [59] (2014) and Niraimathee et al. [60].

The difference in the location of these bands can be connected with the methodology of Fe nanoparticle preparation and their size and shape. The bands in the spectral region 700–1800 cm^−1^ are connected with the organic compounds which have been used to synthesise the nanoparticles.

**Figure 8:**
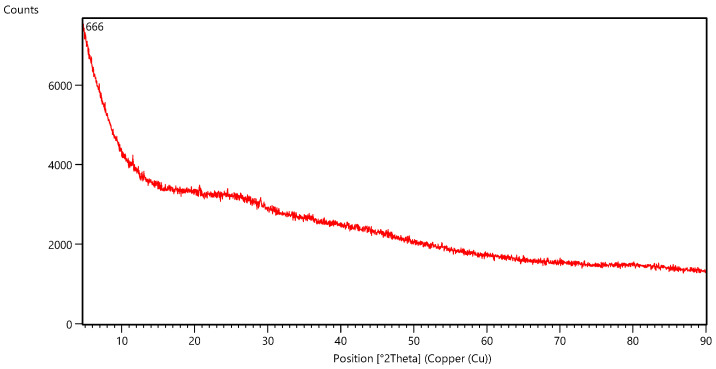
Diffractogram obtained via X-ray diffraction measurement (sample 666). Red line presented intensity of the signal for various angles of diffraction at their respective two theta positions.

Analysing the diffractogram obtained via XRD measurements (Figure 8), no crystalline form of the nanoparticles was observed, probably due to the low concentration of iron in the samples.

Samples with concentrations ranging from 0.41% to 0.92% were selected for measurements; however, these concentrations were insufficient to obtain confirmation of the presence of a crystalline form. No characteristic diffraction peak was observed to indicate the amorphous structure of the nanoparticles. A similar difractogram for zero-valent iron was obtained by Eslami et al. [61] during green synthesis of iron nanoparticles using murine extract.

### 3.7. Calorimetric Measurements

To give a precise description of the physicochemical properties of nanoparticles, we need to determine not only the morphology, size and crystal structure, but also the magnetic response of the system to an external magnetic field. Nanomaterials with magnetic properties are superparamagnetic. This phenomenon involves acquiring magnetic properties by nanomaterials after applying an external magnetic field, and their disappearance after removing the source of the magnetic field. Fragmentation of the material causes the separation of magnetic domains, which significantly reduces the coercivity value. Ferromagnetic materials include such metals as iron, cobalt, nickel and oxides of these metals [62].

Hematite demonstrates paramagnetic properties at temperatures above 956 K. It is weakly ferromagnetic at room temperature and undergoes a phase transition at 260 K to an antiferromagnetic state. Magnetite demonstrates ferrimagnetic properties at room temperature and has a temperature of 850 K. Magnetite particles smaller than 6 nm are superparamagnetic at room temperature, although their magnetic properties depend mainly on the methods used in their synthesis. Maghemite demonstrates ferrimagnetic properties at room temperature and instability at high temperatures and loses its magnetic susceptibility over time [63].

Calorimetric measurements involve placing the tested suspension sample (with suspected magnetic properties) in an alternating magnetic field and simultaneously measuring the temperature of this sample. These measurements are most often performed in nonadiabatic systems due to the simple experimental procedure and low analysis costs.

**Figure 9:**
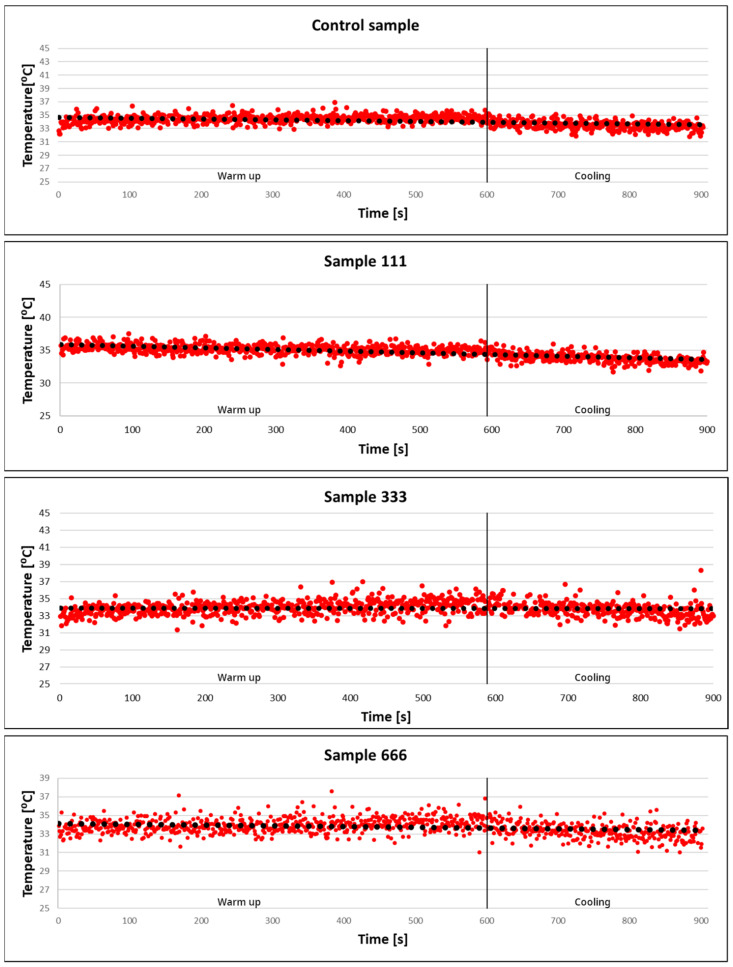
Calorimetric measurements of the control sample and samples 111, 333 and 666 (Red dots are the results of measurements taken every 1 s. The black dots represent the trend line. The vertical black line shows the point at which sample cooling begins.).

As can be seen in the charts (Figure 9), no increase in temperature was observed in any of the samples during the exposure to the electromagnetic field.

Analysing the above charts and literature data, it was found that the resulting nanoparticles do not exhibit magnetic properties under the measurement parameters used. Probably, changing the parameters of the applied external magnetic field and the temperature would cause the particles to become magnetised. Magnetisation is accompanied by an increase in temperature, as described, among others, in the work of Orzechowska et al. [64], Winiarczyk et al. [65] and Rećko et al. [66].

## 4. Conclusions

Green synthesis of iron nanoparticles using various plant extracts has been reported by many researchers. Most often, they have produced iron nanoparticles using green tea extract. In this work, we used strawberry leaves and calyxes for this purpose. These are waste raw materials in agriculture. Analysing the potential of strawberry leaves and calyces as a factor reducing iron precursors, we found that the content of polyphenols and flavonoids in strawberry leaves and calyces as well as their antioxidant activity is high. This confirmed that this raw material may be used as a reducing agent for iron nanoparticle precursors. The reduction of iron ions to iron particles occurred after mixing the precursor with the extract from strawberry leaves and calyxes at a temperature of 20 °C. As described by other authors, the colour of the mixture changed from the colour typical of dissolved salt to black. TEM microscopic analysis and particle distribution confirmed that the size of the obtained iron nanoparticles depended on the precursor concentration. In most observations, the shape of the nanoparticles was close to spherical. Zeta potential analysis confirmed the formation of negatively charged particles. The high iron content in the samples was determined based on XRF analysis. The remaining elements detected during this analysis are characteristic of plant extracts and result from the plant growth physiology. The obtained iron nanoparticles did not demonstrate magnetic properties. In summary, the use of strawberry leaves and calyces enables the production of iron nanoparticles with a size dependent on the precursor concentration, and may serve as an excellent alternative to other methods.

## Figures and Tables

**Table 1:** Precursors of iron nanoparticles used in experiment.

No.	Sample	Precursor	Concentration (mol/dm^3^)
1	1	FeCl_3_ × 6H_2_O	0.5
2	2	Fe_2_(SO_4_)_3_ × H_2_O	0.5
3	11	FeCl_3_ × 6H_2_O	0.1
4	22	Fe_2_(SO_4_)_3_ × H_2_O	0.1
5	33	FeCl_2_ × 4H_2_O	0.1
6	44	Fe(NO_3_)_3_ × 9H_2_O	0.1
7	111	FeCl_3_ × 6H_2_O	0.01
8	333	FeCl_2_ × 4 H_2_O	0.01
9	555	FeCl_3_ anhydrous	0.01
9	666	FeSO_4_ × 7H_2_O	0.01

**Table 2:** The content of phytochemicals in strawberry leaves and calyces and their antioxidant activity.

Total Phenolic Content (mg GAE/1 g)	Total Flavonoid Content (mg QE/1 g)	DPPH (µM TE/1 g)	FRAP (µM TE/1 g)
63.88 ± 3.61	4.66 ± 0.52	346.81 ± 9.51	331.71 ± 36.5

**Table 3:** Examples of plant mediation used to synthesize iron nanoparticles.

Species and Part of Plant	Precursor	Shape	Size (nm)	References
*Avecinnia marina* (flowers)	FeCl_3_	honeycomb	30–100	[45]
*Amaranthus spinosus* (leaves)	FeCl_3_	spherical	58–530	[46]
*Artocarpus heterophyllus* (peel)	FeCl_2_	spherical	33	[44]
*Moringa oleifera* (leaf and seed)	FeCl_3_.6H_2_O	spherical	250–474	[47]
*Punica granatum* (seeds)	FeCl_3_	Semi-spherical	25–55	[48]
*Fragaria × ananassa* (leaves and calyces)	FeCl_3_ × 6H_2_O	spherical	13–34	Present study
	FeCl_2_ × 4H_2_O	spherical	70–145	
	FeCl_2_ × 4H_2_O	variable shape (from nanocubes and spheres to triangles with irregular edges)	50–78	

**Table 4:** Zeta potential values of measured samples.

Sample	Concentration (mol/dm^3^)	Precursor	Zeta Potential Values x ± SD (mV)
Control	-	-	−11.183 ± 0.248 ^d^
1	0.5	FeCl_3_ × 6H_2_O	−17.156 ± 0.900 ^c^
2	0.5	Fe_2_(SO_4_)_3_ × H_2_O	−17.489 ± 0.540 ^c^
11	0.1	FeCl_3_ × 6H_2_O	−17.944 ± 0.725 ^c^
22	0.1	Fe_2_(SO_4_)_3_ × H_2_O	−12.762 ± 1.942 ^d^
33	0.1	FeCl_2_ × 4H_2_O	−21.167 ± 1.074 ^a^
44	0.1	Fe(NO_3_)_3_ × 9H_2_O	−20.900 ± 0.447 ^ab^
111	0.01	FeCl_3_ × 6H_2_O	−21.078 ± 0.792 ^a^
333	0.01	FeCl_2_ × 4H_2_O	−21.300 ± 1.120 ^a^
555	0.01	FeCl_3_ anhydrous	−18.000 ± 1.346 ^c^
666	0.01	FeSO_4_ × 7H_2_O	−18.933 ± 2.722 ^bc^

^a,b,c,d^ Means in the same column indicated by different letters were significantly different (*p* value < 0.05).

**Table 5:** XRF measurement results for selected samples.

Element	Sample							
	Atomic (%)							
	Control	11	22	44	111	333	555	666
Si		0.35						
P	0.2	0.42	0.26	0.21	0.2	0.2	0.3	0.26
Cl	0.23	0.024	0.015	0.007	0.026	0.01	0.19	0.023
K	0.42	0.016	0.01	0.012	0.034	0.032	0.14	0.028
Ca	0.52	0.14	0.13	0.22	0.32	0.2	0.11	0.23
Fe	0.007	1.31	0.96	0.09	0.41	0.13	0.6	0.92
S	0.4		0.34		0.04			
weight of the entire sample (g)	3.47	1.8	1.9	1.87	1.75	1.49	1.88	1.89
% of the sample that can be determined	1.665	2.41	1.847	0.632	1.229	0.668	1.205	1.622

## Data Availability

The original contributions presented in the study are included in the article, further inquiries can be directed to the corresponding author.
